# CHARACTERISTICS OF LONG-TERM HOME CARE PATIENTS WITH DEMENTIA

**DOI:** 10.1093/geroni/igac059.1998

**Published:** 2022-12-20

**Authors:** Takashi Yamanaka, Maiko Mizuki, Kiwami Kidana, Ryonosuke Yamaga, Masahiro Akishita

**Affiliations:** Graduate School of Medicine, The University of Tokyo, Bunkyo-ku, Tokyo, Japan; Graduate School of Medicine, The University of Tokyo, Bunkyo-ku, Tokyo, Japan; Graduate School of Medicine, The University of Tokyo, Bunkyo-ku, Tokyo, Japan; Tsubasa Home Care Clinic, Funabashi-city, Chiba, Japan; Graduate School of Medicine, The University of Tokyo, Bunkyo-ku, Tokyo, Japan

## Abstract

This study aimed to clarify the characteristics of older adults with dementia receiving physician home visits. We prospectively registered 179 patients aged ≥65 years, estimated to receive physician home visits for over 6 months, in a clinic in Chiba, Japan, in 2020–2021. The patients’ mean (±SD) age was 85.1±7.4 years and ranged from 68 to 102 years. Out of the total patients, 57.5% were men. We collected clinical information; employed the Dementia Assessment Sheet in Community-based Integrated Care System 21 items (DASC-21) and EuroQOL five dimensions 5-level (EQ-5D-5L) every 6 months; and calculated the incidence of death. Eighty-two patients (45.8%) were diagnosed with dementia at the commencement of physician home visits. The characteristics of older adults diagnosed with dementia (D+ group) were compared with the characteristics of those who were not (D- group). The D+ group was older than the D- group (86.4±6.6 years vs. 84.1±8.0 years, p=0.04118). Total cholesterol levels and DASC-21 scores were higher in the D+ group compared to the D- group (180.2±41.7 vs. 163.7±51.2, p=0.04091; 62.3±14.5 vs. 50.2±16.2, p=0.00004, respectively). The EQ-5D-5L was not significantly different between the groups (0.439±0.255 vs. 0.397±0.267, p=0.32409). The proportion of those living in assisted living facilities or fee-based homes for the elderly was higher in the D+ group (χ2=8.5177, df=2, p=0.01414, V=0.2258). The mortality rate after 6 months was 20.7% in the D+ group and 16.5% in the D- group (χ2=0.5305, df=1, p=0.46641). In conclusion, the characteristics of patients with dementia should be further elucidated to provide better care.

